# To stay or grow? Migration patterns and child growth in rural Bihar, India

**DOI:** 10.1016/j.healthplace.2024.103395

**Published:** 2025-01

**Authors:** Reshma P. Roshania, Solveig A. Cunningham, Aritra Das, Tanusree Bag, Rakesh Giri, Guntur Sai Mala, Melissa F. Young, Sridhar Srikantiah, Tanmay Mahapatra, Usha Ramakrishnan

**Affiliations:** a Nutrition and Health Sciences, Laney Graduate School, Emory University, Atlanta, GA, United States; b CARE India Solutions for Sustainable Development, Patna, BR, India; c Hubert Department of Global Health, Rollins School of Public Health, Emory University, Atlanta, GA, United States

**Keywords:** Internal migration, Circular migration, Rural, Child growth, India

## Abstract

While the dominant patterns of migration for livelihood among the poor in India are rural-to-rural and circular, literature on the health implications of child migration has largely focused on rural-to-urban, permanent movement. We compared child growth across three migration typologies rural Bihar: circular migrant families that repeatedly migrate to rural destination sites with accompanying young children, rural households with male migrants, and rural households that do not engage in migration. We integrated network theory based on caste and tribe geography to inform our analytical approach. Our results demonstrate complex associations between nutrition status and repeated movement of children between home and destination spaces. In addition to the policy imperative of multilocational strategies for migrant families, households that do not engage in migration yet are located in high outmigration regions also require targeted livelihood and health interventions.

## Highlights

• The health impact of child migration to rural destination sites is lesser studied.
• To understand the impact of migration on children, a counterfactual is necessary.
• We used network theory based on caste and tribe geography to create propensity score weights.
• Recurrent movement between home and destination disconnects children from care.
• Policy should be targeted to non-migrant families in high outmigration localities.

## 1 Introduction

Undernutrition is the underlying cause of nearly half of all child deaths globally (World Health Organization, 2023). India is home to one third of the world's children under five who are chronically undernourished, or stunted, and over one half of the world's children under five who are acutely undernourished, or wasted (Development Initiatives, 2018). Both stunting and wasting reflect nutritional insults from deprivations in children's environments beginning in utero, and which can result in negative consequences in body composition and physiological function (Thurstans et al., 2022). The consequences of undernutrition in childhood extend into adolescence, adulthood, and intergenerationally, with respect to schooling attainment, income, chronic disease risk, and offspring birthweight (Victora et al., 2008). The basic causes of both manifestations of undernutrition include poverty and inequity (UNICEF, 2015), which strongly persist in India. Despite decades of large-scale implementation of nutrition and social protection programming by the state targeted to the poor, the prevalence of stunting and wasting in some states of the country has increased in recent years (International Institute of Population Sciences (IIPS), 2020).

Over 60 percent of the Indian population relies on agriculture for livelihood, either as farmers or farm labour (World Bank, 2007). In India, the majority of land ownership is smallholdings of less than 1 ha, which is insufficient to meet the needs of most households (Shah and Harriss-White, 2011). In this context, outmigration is a critical component of a multiple livelihood strategy undertaken by many rural households in India to cope with poverty.

Labour migration dynamics are highly gendered: 85 percent of labour migrants are men, the greatest proportion of whom work in the service sector (42 percent) (Mazumdar et al., 2013). Among women labour migrants, the greatest proportion (34 percent) migrate for work in agriculture. This pattern is associated with important variations in seasonality and destinations of migrants with implications on household structures at home and during migration. For example, movement of the whole family, including young children, are often rural-to-rural, short-term, circular, and over short distances (Deshingkar and Farrington, 2009). The phenomenon of ‘missing men’ from source villages, on the other hand, arises from substantial migration of unaccompanied male household members to farther urban centres, for longer periods of time.

Child growth can be affected by these gendered migration streams. Non-migrant children with migrant fathers may have improved nutrition due to increased food security, improved diet quality, and access to health care from remittances (Zezza et al., 2011). There is global evidence that remittances improve food security and dietary diversity; yet the impact on nutrition is limited (Thow et al., 2016). National studies from India have found that remittances from migrant family members reduce food insecurity and increase overall food consumption (Rahman and Mishra, 2020; Sangwan and Tasciotti, 2023). Specific to male migration, a study from Bihar found that remittances from male family members increases consumption of nutrient-rich animal source foods, including dairy, eggs, fish and meat (Choithani, 2017).

Conversely, the psychological effects of parental absence, and the reduced time and energy of the remaining caregivers due to additional household responsibilities can have detrimental effects on child health. A multi-country study of Ethiopia, India, Peru and Vietnam, found that, while per capita consumption increases, children in households with a migrating parent have worse anthropometric and cognitive outcomes compared to children in non-migrant households (Nguyen, 2016). Research from India specific to male migration finds that the effect of male migration on child nutrition outcomes is moderated by gender and community contexts (Lei et al., 2020).

Children who accompany their parents who migrate for work may benefit by improved diets from increased income, but unhygienic living conditions in many places of destination may worsen health and nutrition outcomes. Children who move are also disconnected from social protections such the Integrated Child Development Services (ICDS), the national program in India that supports both early childhood nutrition and development. Literature on migration and child health in India has primarily focused on children of rural-to-urban, permanent migrants. These studies suggest that compared to urban non-migrant children, children of rural-to-urban migrants are less likely to be immunized, more likely to be undernourished (Kumar et al., 2016; Prusty and Keshri, 2015), and face a higher risk of mortality (Stephenson et al., 2003). Food security and access to nutritious foods is also worsened at urban destination sites due to high food prices, the loss of food entitlements, and the high costs of rent and children's education (Rai and Selvaraj, 2015). Duration of migration and political contact are important considerations; long-term migrants in urban environments represent a politically relevant group, and can eventually obtain access to health care (Kusuma et al., 2013), water and sanitation services, and Public Distribution System (PDS) rations – the main pillar of India's food security entitlements (Edelman and Mitra, 2006). Migration in India, however, is dominated by short-term, circulatory patterns, and as mentioned above, for accompanying children, rural-to-rural movement. Destination sites of those engaging in family circular migration, such as brick kilns and farms, are often geographically isolated, and circular migrants are politically disconnected in their places of work.

Moreover, comparing migrant children to non-migrant children in the place of destination does not provide insights into the impact of migration on children. The critical inquiry is how children who move with their families would nutritionally fare if they remained in the origin; this question has not been answered and requires a comparison of children who move with those in source villages. Such research can enable a better understanding of migration outcomes, which has long been lacking in policy discourse in India (Deshingkar, 2017).

The objective of this study is to compare nutrition status among circular migrant children in rural destination spaces with rural non-migrant children. Among non-migrant children, we differentiate those in households that do not engage in any migration from those in households with male migrant members. There have been no studies to our knowledge that compare children who move with children who remain in male migrant sending households.

## 2 Theory and study context

There is substantial variability throughout India with respect to poverty, agrarian dynamics, migration outflows, and child nutrition. We conducted our study in the state of Bihar, the third most populous state in India, and the poorest (IIPS, 2017). Bihar is also the least urbanized; 84 percent of the population lives in rural areas (IIPS, 2021), largely relying on farming for livelihood. Agricultural performance of the state is poor (Kumar and Maulick, 2016); small landholdings, lack of irrigation infrastructure and climate shocks all contribute to very high temporary outmigration from the state. Other states that are similar to Bihar with respect to rural livelihood and human development include Uttar Pradesh, Rajasthan, Chhattisgarh and Jharkhand.

Circular migration streams in Bihar, similar to the rest of the country, fall along axes of social groupings; those in marginalized communities including Scheduled Castes (SC) and Scheduled Tribes (ST) are the most likely to engage in precarious forms of movement – informal, and through labour contractor intermediaries (Deshingkar and Farrington, 2009). While the contractor is often seen as an exploitative actor who traps families in debt by recruiting labour against cash advances, this person is also viewed as the only route to stable work opportunity and loans in the absence of formal banking access (Deshingkar, 2017). Based on the importance of the role of the labour contractor to circular migration dynamics, we draw from recent scholarship on migration networks - specifically, demand (i.e., employer) driven recruitment through employee referrals (Iversen et al., 2009).

Basic network theory postulates that migration from one area facilitates further migration from that area based on information flows and kinship ties (de Haas, 2010), which places those who have already migrated and those who may potentially migrate as the key players in the decision process. However, including the employer at the destination is an important demand-side factor in network effects. Especially in lower skilled industries, employers are incentivized to leverage employees’ social networks to avoid workplace moral hazard (Dhillon et al., 2021). That is, recruits who are referred by employees in the same social network will be more likely to perform well due to strong ties with the referee. We expand the category of referees to include labour contractors, as they are usually from the same geographic and social communities as labourers (Gupta, 2003), and increasingly relevant, worker-agents (manual labourers who supplement their earnings by recruiting other workers for a small commission (Raj and Axelby, 2019)). Thus, rather than the social group of the individual, it is imperative to consider the caste and tribe distribution of a geographic community as a predictor of migration. To integrate this theoretical principle in our research, we define and match on high and low outmigration districts based on SC and ST population sizes, as further described below in the methods section.

To study nutrition among children who engage in circular migration with their families, we focused our research within the brick industry. Brick kilns throughout South Asia utilize traditional processes for brick manufacturing, relying on manual, low-skilled labour. Due to the practice of recruiting male-female pairs, family units - commonly including young children - migrate and live on-site in rudimentary housing for the duration of the season. Brick kilns operate seasonally during the dry months (generally October to June in Bihar), and are mainly located in rural and peri-urban areas. As mentioned above, labour contractors provide advances to workers before the operating season begins; this amount, plus weekly allowances that families receive during the season for food and other costs such as healthcare, is deducted from the families’ earnings, which are withheld until the end of the season. Brick kilns are the predominant destination site for rural-to-rural, circular migration undertaken by nuclear family units; it is estimated the industry employs 15 million workers in India, most of whom are migrants (Eil et al., 2020). While focusing our study within the brick kiln context can provide important insights into the rural-to-rural family circular migration stream, it is important to note that our study is not representative of all rural-to-rural family circular migrants. For the purposes of this manuscript, we will henceforth refer to our study population of circular migrant families to brick kilns in Bihar as circular migrant families.

### 2.1 Conceptual framework

**Fig. 1:**
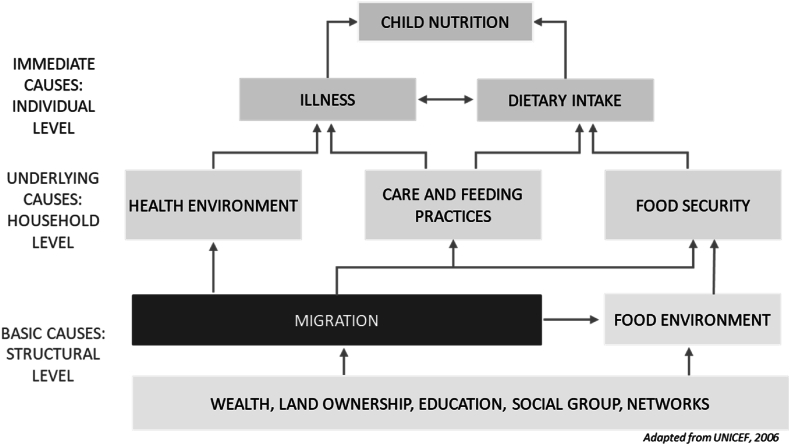
Conceptual framework of migration and the determinants of child nutrition.

We adapted the UNICEF Conceptual Framework of the Determinants of Child Undernutrition (UNICEF, 2015), which has been used widely as the theoretical basis for global nutrition intervention design and implementation. The framework outlines the primary pathways among the basic, underlying, and immediate determinants of child malnutrition. The decision to migrate and the type of migration (e.g. family versus male only migration, rural-to-rural versus rural-to-urban migration, etc.) is a response to the structural level inequities that drive disparities in the underlying and immediate causes of malnutrition, and ultimately nutrition status and its consequences. Specifically, we conceptualize migration type to be shaped by the basic causes of undernutrition, including wealth, education, social group, and land ownership, i.e. the structural determinants of child nutrition. These factors influence the underlying determinants at the household level - access to health care, water and sanitation, caregiving and food security, which in turn determine illness and diet, the immediate causes of child malnutrition at the individual level. Critically, the inclusion of migration as a structural determinant highlights that the outlined causal pathways are not static; this is particularly the case for circular migrant families, for whom household food security, access to health care, feeding practices, and dietary intake can vary periodically over time *and* geographies. This framework, shown in Fig. 1, facilitates an exploration of the potential ways migration operates in influencing nutrition status. We use this adapted framework to identify important variables of interest described in section 3.2.2 that may differ among children who circularly migrate with their families, children in male migrant households, and children in non-migrant households.

## 3 Data and methods

### 3.1 Data sources

This study was conducted by CARE India and Emory University. We combined data from two different surveys implemented by CARE India in Bihar during the same time period.

#### 3.1.1 Children who accompany their families during circular migration

In June 2018 we conducted a multi-stage stratified cluster design survey across 519 randomly selected brick kilns (clusters) throughout 37 of the 38 districts (strata) with operational brick kilns in Bihar. Kilns were randomly selected from the district lists of active kilns maintained by the Government of Bihar Department of Mines and Geology. Based on sample size calculations to detect a subgroup difference in stunting prevalence and a cluster size of three, explained below, we sampled eighteen kilns within each strata; for each sampled kiln, we identified eligible circular migrant households residing on-site based on the following criteria: 1) self-identification as a circular migrant household, defined as living away from their home block (sub-district) for employment purposes for a total of at least 60 days in the previous year, with at least one return home during that year; and 2) presence of at least one child under three years of age at the kiln. We first conducted a census to register and generate a list of all circular migrant families meeting the eligibility criteria listed above; from the listing thus generated, we randomly selected three households, one with a child 0–11 months of age, and two with at least one child 12–35 months of age. If the selected family had multiple children under three years of age, information was collected for the youngest child. The cluster size of three was determined based on formative research that indicated around five to ten migrant children under three are likely to be present on a given kiln. A household survey questionnaire was administered to the mother of the selected child, and height/recumbent length and weight data were collected for each selected child.

**Fig. 2:**
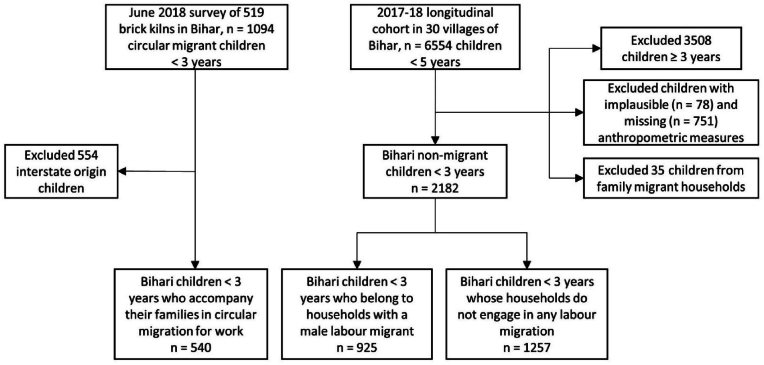
Flow chart of participant selection for analysis.

We collected data on 1094 children under three in June 2018; we restricted the analysis to the subset of children whose state of origin is Bihar (n = 540) (Fig. 2). Details on methods for this survey have been previously published (Roshania et al., 2022).

#### 3.1.2 Children from male migrant and non-migrant households

From August 2017 to July 2018, CARE India implemented a longitudinal cohort study to determine the incidence of severe acute malnutrition (SAM). Ten districts were chosen, one from each of the ten CARE India programmatic regions in Bihar. Within the ten districts, three villages were purposively selected based on low, medium, and high accessibility to the district capital. All children under five years of age from the selected 30 villages were enrolled into the open cohort. A household survey was administered to the caregiver of each child once during the study period. The instrument included a migration module, which identified members of the household who migrated for work for at least two months in the previous one year. Weight data and information on illness in the previous month for each child was collected monthly, and height/recumbent length data was collected once every three months. We interpolated weight data for the interval months based on the preceding and subsequent weight measures.

The cohort consisted of 6554 children under five. For this analysis, we restricted the data to children under three years of age for whom weight and height (measured or interpolated) were available for June 2018. We subset the analysis to children whose fathers migrated out (n = 925) and households that did not engage in any migration during the previous one year (n = 1257) (Fig. 1).

All data were collected digitally using handheld tablets by CARE India staff who were trained on the survey instrument and anthropometric measurement.

### 3.2 Outcomes of interest

#### 3.2.1 Primary outcomes

Our primary outcome of interest was stunting and wasting, defined as a height-for-age and weight-for-height z-scores, respectively, of less than negative two standard deviations. Z-scores were calculated using World Health Organization references (WHO Multicentre Growth Reference Study Group, 2006).

#### 3.2.2 Secondary outcomes

We used the UNICEF Conceptual Framework of the Determinants of Child Undernutrition (UNICEF, 2015) described above to determine secondary outcomes of interest. At the level of underlying determinants, we measured food insecurity in the previous year using the Food Insecurity Experiences Scale (Cafiero et al., 2018). Household food availability was measured by asking the respondent how many days of the previous week food groups were available; a predetermined list, developed by CARE India, of 31 food groups commonly consumed in the region was used. Data on primary source for drinking water and access to sanitation was also collected. Access to the public health system was assessed by asking if the child ever received any immunization, ever received iron supplementation for children six months and older, and received deworming medicine in the last six months for children 12 months and older. Among the immediate determinants, we measured illness in terms of diarrhoea and acute respiratory infection (ARI) symptoms in the previous one month. Current breastfeeding among children under six months, timely initiation of complementary feeding among children six to eight months and consumption of a minimum acceptable diet among children 6–23 months were used to measure infant feeding practices (World Health Organization, 2010).

### 3.3 Propensity score weighting

Circular family migration is selective, in that families who circularly migrate are different from families who do not engage in migration in many important aspects that also impact child nutrition, such as wealth, land ownership, parental education, and social group. Controlling for variables in a regression analysis without addressing covariate imbalance can lead to model dependence and imprecise estimates (Ho et al., 2007). Thus, to create groups of comparable children with respect to important factors that predict migration, we conducted propensity score weighting to ensure adequate balance. We weighted on the following covariates: wealth quintile, which was calculated using principal component analysis methods of household asset ownership data; land ownership (dichotomous); paternal education (any/none); child age in months; child sex; and proportion of district population that are SC or ST. For district SC and ST proportion, we used data from the Socio Economic and Caste Census 2011 (Ministry of Rural Development, 2011) to classify each district in Bihar as high SC and ST (combined proportion of SC and ST greater than 20 percent) or low SC and ST (combined proportion of SC and ST less than or equal to 20 percent). For circular migrant families, district of origin was used for classification and matching.

We utilized the multiple groups covariate balance propensity score (CBPS) (Imai and Ratkovic, 2014) methodology to generate propensity scores and corresponding weights. CBPS optimizes covariate balance and prediction of treatment assignment; in this case, propensity scores represent the likelihood of engaging in family migration. Weights were generated and applied to observations from non-migrant and male migrant households, so that children from these groups looked similar to circular migrant children on the specified covariates; weights for circular migrant children had a value of one. In other words, after weighting, non-migrant and male migrant households represented groups that had a high propensity to migrate as a family unit, but did not engage in migration. We achieved standardized mean differences (SMD) of less than 0.1 for all variables after weighting, which is considered an acceptable threshold (Imai and Ratkovic, 2014). The effective sample sizes (ESS) of family circular migrant children, children in male migrant households, and children in non-migrant households after applying weights were 540, 121, and 123, respectively.

### 3.4 Statistical analysis

We first conducted descriptive statistics to explore differences in sociodemographic characteristics by family migrant, male migrant and non-migrant households prior to applying CBPS weights; we used chi-square for categorical variables and analysis of variance for continuous variables to test for significant differences among the three groups. We then conducted weighted bivariate analyses between migrant group and the secondary outcomes of interest (i.e. the determinants of nutrition status at the basic, underlying, and immediate levels). To obtain weighted odds ratios for the association of migration group with nutrition status, we ran separate logistic regression models with stunting and wasting as outcomes and migration group as the exposure of interest. To explore if the migration group and nutrition pathways outlined in the conceptual framework potentially differ between males and females, we checked for interaction of migration group and child sex; since there was no evidence of interaction, the final models adjusted for age in months and child sex. Alpha was set at 0.05. All analyses were conducted in R and SAS. The WeightIt (Greifer, 2021b) and cobalt (Greifer, 2021a) packages in R were used to generate propensity score weights and balance diagnostics.

## 4 Results

### 4.1 Population characteristics

Sociodemographic characteristics differ by migrant type (Table 1). At the time of the study, family migrants had been residing on the kiln where they were enumerated for an average of 6.5 months. Family circular migrants who migrate to work in brick kilns are more likely to originate from districts with a high proportion of SC and ST, less likely to own land, and are more likely to be uneducated and from the poorer wealth quintiles compared to households from the program districts who did not migrate or have male migrant members. Family circular migrants are also less likely to have a PDS ration card compared to the other migrant groups, and among those who do, are more likely to have the Antyodaya card (issued to the poorest of the poor).

**Table 1:** Population characteristics by migration group, Bihar 2018.

	Non-migrant HH *n=1257*	Male migrant HH *n=945*	Family migrant HH *n=540*
	n (%)	n (%)	n (%)
**High district SC & ST population**^a^	306 (24.3)	286 (30.9)	405 (75.0)

**Land ownership**^a^	618 (49.12)	436 (47.1)	124 (23.0)

**Wealth quintile**^a^			
Lower	123 (9.8)	107 (11.6)	314 (58.1)
Second	215 (17.1)	183 (19.8)	147 (27.2)
Middle	275 (21.9)	219 (23.7)	50 (9.3)
Fourth	293 (23.3)	232 (25.1)	21 (3.9)
Higher	351 (27.9)	184 (19.9)	8 (1.48)

**PDS card**^a^	776 (61.7)	595 (64.3)	245 (45.4)

**PDS card type**^a^**(n = 1616)**			
Above poverty line	162 (20.9)	109 (18.3)	26 (10.6)
Below poverty line	470 (60.6)	365 (61.3)	139 (56.7)
Antyodaya	69 (8.9)	44 (7.4)	37 (15.1)
Annapurna	56 (7.2)	50 (8.4)	17 (6.9)
Don't know	19 (2.5)	27 (4.5)	26 (10.6)

**Household size, mean (sd)**^a^	7.4 (3.3)	7.0 (3.2)	6.4 (2.8)

**Father's education (any)**^a^**(n = 2695)**	630 (50.4)	460 (49.7)	71 (13.7)

**Mother's education (any)**^a^	421 (33.5)	305 (33.0)	26 (4.8)

**Child sex (female)**	610 (48.5)	453 (49.0)	261 (48.3)

**Child age (months), mean (sd)**^a^	19.3 (9.5)	19.7 (9.9)	16.3 (9.0)

Cells contain column percentages.

Abbreviations: SC – Scheduled Caste; ST – Scheduled Tribe; PDS – Public Distribution System; sd – standard deviation.

Antyodaya PDS cards are issued to the poorest of the poor. Annapurna PDS cards are issued to poor senior citizens (65 and over).

a p < 0.05.

After calculating and applying propensity score weights, adequate balance among the three migrant groups was achieved on district SC and ST population, wealth quintile, land ownership, paternal education, child sex, and child age. SMD before and after weighting are summarized in the Appendix.

### 4.2 Child growth

**Fig. 3:**
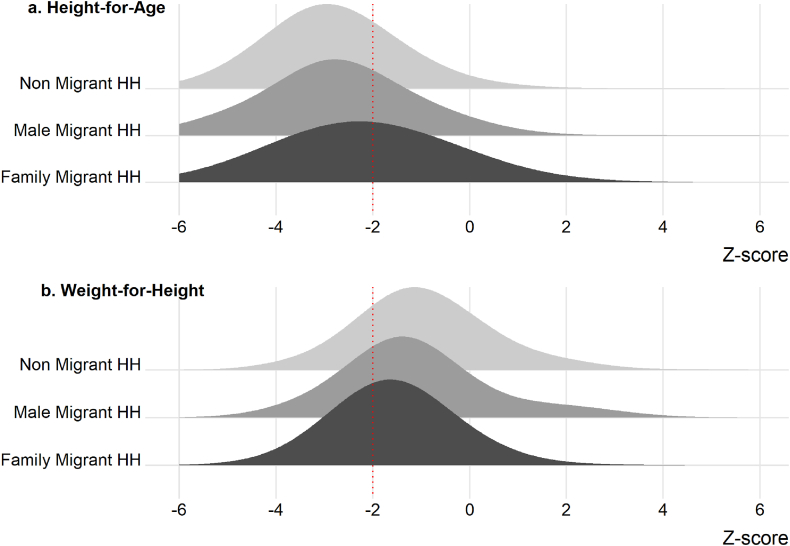
Weighted height-for-age (a) and weight-for-height (b) z-score distributions by migration group, Bihar 2018 The red dotted line at −2 z-score represents the threshold for classifying stunting and wasting.

Overall median HAZ and WHZ were −2.7 and −1.3, respectively. Fig. 3 shows the weighted distributions of HAZ and WHZ by migration group. HAZ distributions (3a) of child migrants are shifted to the right compared to non-migrant children and children in households with male migrants, indicating a lower prevalence of stunting among children who migrate with their families. Overall stunting is 77.8 percent among children under three in non-migrant households, 73.7 percent among children in households with male migrants, and 55.0 percent among circular migrant children. WHZ distributions are shifted to the left for child migrants compared to the other migrant groups (3b), indicating a higher prevalence of wasting among children who migrate. Overall wasting is 18.6, 24.0 and 34.1 percent, among children in non-migrant, male migrant and family migrant households, respectively.

The adjusted odds ratio estimates of stunting and wasting reflect the z-score distributions. Adjusting for age and sex, children in non-migrant households that are similar to family circular migrant households are almost three times as likely to be stunted but 55 percent less likely to be wasted compared to children who engage in circular migration. Children in households with a male migrant member are over twice as likely to be stunted and 40 percent less likely to be wasted compared to circular migrant children (Table 2).

**Table 2:** Age and sex adjusted estimates of the association between migration type and nutrition status

	Stunting	Wasting
	OR (95% CI)	OR (95% CI)
**Migration type**		
Family migrant HH	Ref	Ref
Non-migrant HH	2.79 (1.87–4.16)	0.45 (0.27–0.73)
Male migrant HH	2.26 (1.51–3.40)	0.59 (0.37–0.95)

**Child age (months)**	1.03 (1.01–1.05)	1.01 (0.99–1.02)

**Child sex (Female)**	0.82 (0.59–1.15)	0.90 (0.60–1.36)

Abbreviations: HH – household; OR – odds ratio; CI – confidence interval.

**Fig. 4:**
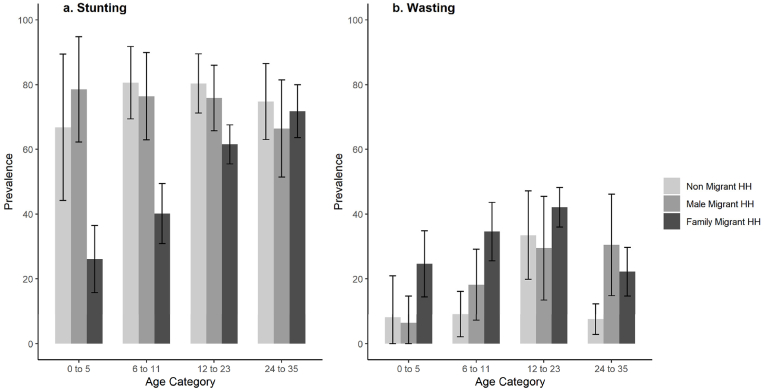
Stunting (a) and wasting (b) prevalence by age category and migration group, Bihar 2018.

Stunting and wasting prevalence by age group is shown in Fig. 4. Under one year of age, the prevalence of stunting among migrant children is substantially lower than the prevalence among the other two migrant groups, whereas after one year, the prevalence among all three groups are similar.

### 4.3 Determinants of nutrition status

**Fig. 5:**
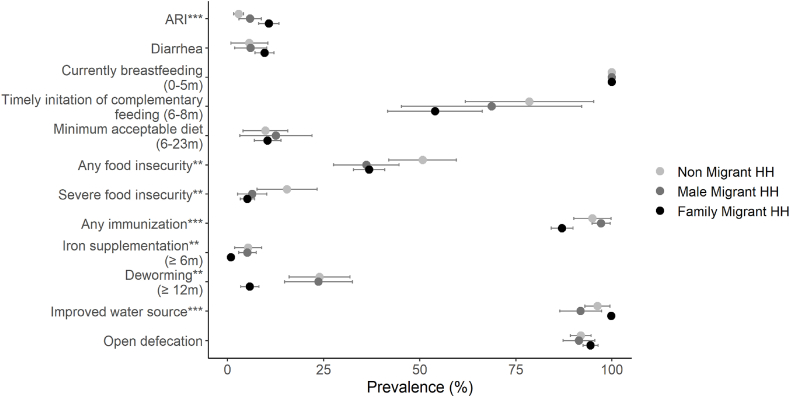
Illness, feeding practices, food insecurity, community-based health coverage, and water and sanitation indicators by migration group, Bihar 2018 ∗∗∗p < 0.001; ∗∗p < 0.01; ∗p < 0.05.

Children who migrate and live on brick kilns are more likely to have had ARI in the previous month (eleven percent) compared to children from male migrant and non-migrant households (six percent and three percent, respectively); there is no difference in diarrhoea prevalence (Fig. 5). There are also no significant differences among the three migration groups in terms of feeding practices – current breastfeeding among children under six months of age (100 percent among all groups), timely initiation of complementary feeding among children six to eight months, and minimum acceptable diet among children 6–23 months. Circular migrant families are less likely to experience any food insecurity in the previous year compared to households that didn't engage in any migration. Access to community-based health and nutrition interventions delivered by front-line health workers is lower among children in circular migrant households compared to male migrant and non-migrant households; this includes ever receiving any immunization, ever receiving iron supplementation, and receiving deworming medicine in the previous six months. Access to an improved water source is slightly higher among migrant families during migration, whereas open defecation was similar across all groups, over 90 percent.

Coverage by the primary public health system community-based interventions is low, and the majority of respondents across all groups report seeking healthcare from the private system. Among children who had ARI or diarrhoea in the previous month, 87% of children from non-migrant households, 95% of children from households with male migrants, and 95% of migrant children were taken to a private health provider, including pharmacists and registered medical practitioners (data not shown).

**Fig. 6:**
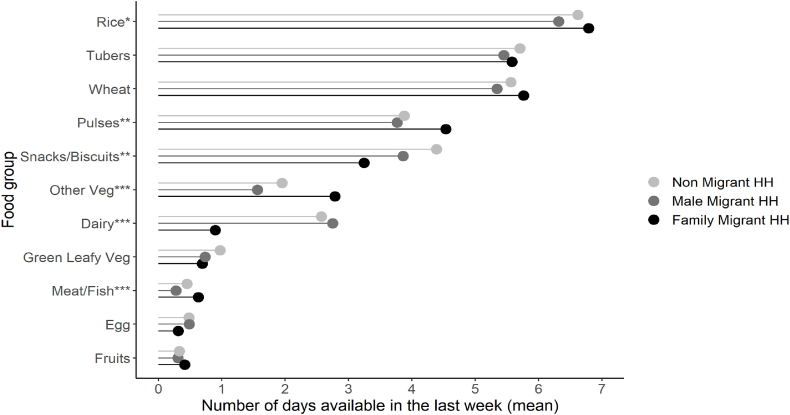
Household food availability by migrant group, Bihar 2018 *∗∗∗p < 0.001; ∗∗p < 0.01; ∗p < 0.05*.

Household food availability in the previous week is displayed in Fig. 6. Among all migrant groups, rice, tubers and wheat are available almost every day of the previous week on average, whereas green leafy vegetables, meat/fish, egg, and fruits are available less than one day of the week on average. Migrant families are more likely to have pulses, vegetables, and meat/fish, and less likely to have dairy available in the previous week compared to non-migrant households and households with a male migrant member.

## 5 Discussion

This study explored the impact of migration on children. We compared the nutritional status of children who accompanied their families in circular migration to the counterfactual – i.e. children who remained in their village of residence but whose households have a high propensity to engage in family circular migration based on wealth, land ownership, father's education, age, sex, and district SC and ST profile. Children in circular migrant families were about one-third as likely to be stunted compared to matched children from non-migrant households. Circular migrant children were over twice as likely to be wasted, however, compared to similar children from non-migrant households. In the context of the growing body of research on the relationship between stunting and wasting (Thurstans et al., 2022), these divergent findings raise important considerations on the specific nutritional pathways that are potentially influenced by seasonal migration and the associated changes in environment.

During migration – which may be up to eight to ten months of the year for work in the brick industry – migrant children appear to have more frequent access to certain nutritious foods such as meat/fish, pulses and vegetables, compared to similar non-migrant children. The profile and bioavailability of nutrients found in animal source foods, namely high-quality protein and zinc, is particularly important for linear growth processes (Michaelsen et al., 2009). Food security was also higher among migrant households compared to similar non-migrant households. This is likely due to regular access to cash, a characteristic of the payment system in the brick kiln and other industries; indeed, in our related qualitative analyses of the changes in food environments between home and destination, circular migrant respondents working in brick kilns overwhelmingly shared that food was more affordable during migration because of regular cash allowances for food purchases (Roshania et al., 2023). Improved dietary quality and quantity for extended periods of time may be one explanatory factor in the observed differences in chronic undernutrition among migrant groups, particularly in early life. Stunting patterns among migrant children were typical in that prevalence was lower among infants and increased with age category, whereas stunting among similar children in non-migrant and male migrant households was very high among all age categories. This is potentially explained by improved maternal dietary intake among women who engage in circular migration which would prevent intra-uterine growth failure and subsequent stunting among infants. By two years of age, however, stunting prevalence among migrant children was just as high as among non-migrant and male migrant children, around 70%. It is important to note that while stunting was the lowest among children who migrate with their families, the prevalence of stunting is high for all three groups of children in our study compared to overall Bihar estimates for the same age group. 37.5 percent of rural children 0–35 months are stunted in Bihar (IIPS, 2021) compared to our overall estimate of 68.7 percent among children in rural households that migrate or have a high propensity to migrate.

Migrant children in our study were less likely to have received services from the community-based health system compared to children who were residing in their home villages. Growth monitoring and the detection, treatment, and referral of acute malnutrition cases is carried out by Anganwadi workers, the ICDS front-line health workers; migrant children living on brick kilns and construction sites are not generally reached by this system, possibly contributing to prolonged acute malnutrition resulting in a higher prevalence of wasting among migrant children. There also may be factors specific to the brick kiln setting that increase the *incidence* of wasting. We observed that migrant children were more likely to experience ARI symptoms in the previous month compared to non-migrant children. In addition to being a leading cause of child mortality, ARI can result in decreased appetite, additional nutrient demand, and malabsorption of nutrients, resulting in a loss of muscle mass. Exposure to particulate matter pollutants is widespread on brick kilns, specifically clay dust from the process of moulding, drying, and carrying bricks, as well as smoke from the burning of coal and biomass needed to fire bricks. Previous studies conducted in brick kilns in India and Pakistan have reported high respiratory illness among laborers (Monga et al., 2012; Shaikh et al., 2012; Thomas et al., 2014). Young children living on brick kilns, although not working, are nonetheless exposed to these pollutants, as are pregnant women. In utero and early life exposure to ambient air pollution has been linked to growth failure in South Asia (Goyal and Canning, 2018; Spears et al., 2019). Our study did not explore air quality, but this is a research need (Sinharoy et al., 2020).

Our findings suggest that overall, some circular migration streams can enable nutritional advantages in dietary intake for children and potentially also mothers, compared to members in non-migrant and male migrant households *like them* in terms of wealth, land ownership, and father's education. In other words, among households who are most likely to engage in precarious streams of migration - that is, poor, lesser educated, landless and from districts with high populations of marginalized groups, those who move as a family are possibly better off with respect to stunting than they may have been had they not migrated for livelihood. However, despite this advantage, characteristics of many destination environments such as limited access to adequate sanitation and essential health services such as immunization can result in increased susceptibility to infection, including diarrhoea, and consequently wasting. Repeated episodes of wasting can result in linear growth failure (Schoenbuchner et al., 2018). These nutritional insults, beginning early in life and accumulative over repeated migrations, may therefore increase the risk of later stunting. Indeed, our study on the association of early life and repeat migration with child nutrition status found that among children who first experienced migration from birth or in the first 6 months of life, those who experienced multiple migrations were 1.6–2.1 times more likely to be stunted compared to those who were experiencing their first migration (Roshania et al., 2022).

Our findings indicate the need to better understand nutrition dynamics among circular migrants between migration episodes, in the place of origin. For instance, debt is a key driver of migration, particularly in industries such as brick manufacturing, where labour contractors provide an advance sum to households which is then paid off during the course of the season; sometimes indebtedness to the kiln owner at the end of the season is what continues the cyclical pattern of migration. Debt can also arise from medical emergencies, wedding costs, agricultural expenses, etc. Our study, like many, used a single time measure of asset ownership to represent wealth, which is inadequate in understanding the fluctuations of income and debt that households experience in between migration cycles. These fluctuations are important drivers of both circular migration and maternal and child nutrition.

With the exception of food security, our results suggest that children from male migrant households are largely similar to children from non-migrant households with respect to nutrition status and determinants of nutrition. However, these findings may result from low ESS of both groups after propensity score weighting. Additional limitations to our study include the exclusion of important covariates, such as maternal nutrition status and other possible sources of unobserved confounding. Due to the cross-sectional nature of the study, we are unable to assess whether nutrition status preceded migration. In other words, it is a possibility that households with children that have a normal height-for-age are more likely to migrate. However, the majority of circular migrant households in our study (68 percent) had been migrating for three or more years, since before the birth of the index child. Lastly, we did not adjust for sampling design in this study because different sampling approaches were used for the two datasets that were pooled. Sampling error thus may not have been adequately accounted for, and as a result standard errors may have been underestimated.

This is the first analysis to compare nutrition among circular migrant children to similar children from non-migrant and male migration households in origin areas; by using propensity score weighting methods, we were able to ensure comparability among all three groups. The setting of Bihar is particularly suitable as it is a state with high poverty, agricultural distress, undernutrition, food insecurity, and the greatest outmigration in the country. Similarly brick kilns represent a major rural destination for family circular migrants; however, future research should explore nutrition dynamics in other industries, such as sugarcane. This study makes a notable contribution to the conceptual framing of child nutrition research and interventions. For countries that experience high family circular movement such as India, it is critical to consider how migration influences underlying and immediate determinants of nutrition, as migration is often driven by the same structural inequities that result in malnutrition including poverty, undereducation, and social discrimination. The pathways connecting the determinants of nutrition status at all levels are thus variable over time and place. In other words, for children who recurrently move, health and food environments, food security, feeding practices, dietary intake, and illness are in a state of flux. Furthermore, the experience of migration may work positively for some pathways, such as food security, but negatively for others, such as healthcare access.

Our findings have implications for policies pertaining to migration and social protection. In the context of the decline of the pro-poor welfare state over previous decades, household wealth represents increasing importance for child nutrition (Chalasani and Rutstein, 2014). Migration for livelihood can enable nutrition security for households, and should thus be facilitated by enforcing labour policies that outline fair pay, safety standards, and child care. Risks to nutrition brought about by migration should be prevented through multilocational programs targeted to migrant families while they are in their home and destinations.

Simultaneously, it is imperative to note that coverage of the public health outreach system is very poor among children in non-migrant and male migrant households that are similar to circular family migrant households. For example, only around five percent of children in both non-migrant and male migrant households ever received iron supplementation in our study, compared to the higher, albeit suboptimal, 22 percent state-wide average of children 6–59 months who received iron supplementation in the previous one week (Rai et al., 2021). Similarly, while open defecation was close to 95 percent among circular migrant families at their place of destination, 91 percent of non-migrant and male migrant households reported open defecation in our study. This is compared to around half of the rural population who are estimated to defecate in the open at the end of the Swachh Bharat Mission in Bihar (Spears et al., 2021). Households that don't engage in migration but face the same socioeconomic vulnerabilities as circular migrant households such as poverty, landlessness, lack of education, and social discrimination are poorly reached by government health services despite not engaging in repeated movement.

Lastly, existing policy recommendations regarding internal migration are largely oriented towards addressing systematic exclusion of migrants from urban spaces (Behera, 2018; International Labor Organization, 2020; Ministry of Housing and Urban Poverty Alleviation, 2017). Our findings illustrate the gaps in entitlements and protections among families who engage in rural-to-rural migration; policy efforts must also explicitly consider social protection inclusion of migrants in rural destination environments, which often are less regulated and more disconnected from the health system. For example, we found that among both migrant and non-migrant caregivers of children who experienced illness in the previous month, the vast majority sought care in the private sector. Indeed, the private health sector accounts for 75 percent of health services in the country (Kumar and Prakash, 2011); most healthcare in rural India is provided by practitioners with no formal training (Mohanan et al., 2016). For circular migrants in their place of destination, the risks of utilizing rural medical practitioners can be exacerbated by language barriers, discrimination, and the fact that in the brick industry as we observed, kiln owners and managers often are the gatekeepers to healthcare in migrants’ unfamiliar destination settings. Health policy targeted towards rural-to-rural migrants must therefore address the challenges of the rural healthcare system.

In conclusion, we found complex associations between child nutrition and circular migration patterns, indicating that migration of children has some nutritional advantages, particularly with respect to food security and dietary diversity. Engaging in migration also implies some nutritional risks, namely exposure to unhealthy environments and decreased access to health services while away from home. Programmatic and policy efforts should adopt approaches to improve nutrition among circular migrant children that recognize the multifaceted ways circular migration can influence nutrition, and must also prioritize households in rural India that experience similar class, education and social group vulnerabilities as circular migrant households but do not engage in migration.

## CRediT authorship contribution statement

**Reshma P. Roshania:** Writing – review & editing, Writing – original draft, Formal analysis, Conceptualization. **Solveig A. Cunningham:** Writing – review & editing, Supervision. **Aritra Das:** Writing – review & editing. **Tanusree Bag:** Data curation. **Rakesh Giri:** Investigation. **Guntur Sai Mala:** Project administration. **Melissa F. Young:** Writing – review & editing. **Sridhar Srikantiah:** Supervision, Conceptualization. **Tanmay Mahapatra:** Supervision, Conceptualization. **Usha Ramakrishnan:** Writing – review & editing, Supervision, Conceptualization.

## Disclosure statement

The authors report there are no competing interests to declare.

## Funding

This research was supported by 10.13039/100000865Bill and Melinda Gates Foundation under Grant OPP1084426.

## Figures and Tables

**Table:** 

	Maximum SMD before weighting	Maximum SMD after weighting
**High district SC & ST population**	0.51	0.04
**Land ownership**	0.26	0.03
**Wealth quintile**		
Lower	0.48	0.05
Second	0.10	0.04
Middle	0.14	0.01
Fourth	0.21	0.00
Higher	0.26	0.00
**Paternal education**	0.37	0.01
**Child sex (female)**	0.01	0.05
**Child age (months)**	0.36	0.06

Abbreviations: SMD – standardized mean difference; SC – Scheduled Castes; ST – Scheduled Tribes.

## Data Availability

Data will be made available on request.
